# Recent Developments in Quantitative Graph Theory: Information Inequalities for Networks

**DOI:** 10.1371/journal.pone.0031395

**Published:** 2012-02-15

**Authors:** Matthias Dehmer, Lavanya Sivakumar

**Affiliations:** Institute for Bioinformatics and Translational Research, UMIT, Hall in Tyrol, Austria; Queen's University Belfast, United Kingdom

## Abstract

In this article, we tackle a challenging problem in quantitative graph theory. We establish relations between graph entropy measures representing the structural information content of networks. In particular, we prove formal relations between quantitative network measures based on Shannon's entropy to study the relatedness of those measures. In order to establish such information inequalities for graphs, we focus on graph entropy measures based on information functionals. To prove such relations, we use known graph classes whose instances have been proven useful in various scientific areas. Our results extend the foregoing work on information inequalities for graphs.

## Introduction

Complexity is an intricate and versatile concept that is associated with the design and configuration of any system [1], [2]. For example, complexity can be measured and characterized by quantitative measures often called indices [3]–[5]. When studying the concept of complexity, information theory has been playing a pioneering and leading role. Prominent examples are the theory of communication and applied physics where the famous Shannon entropy [6] has extensively been used. To study issues of complexity in natural sciences and, in particular, the influence and use of information theory, see [7].

**Figure 1:**
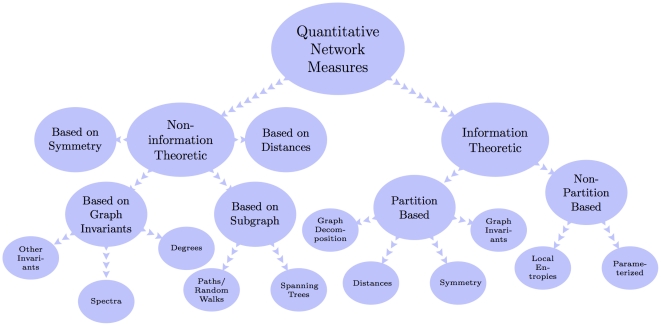
A classification of quantitative network measures.

In this paper, we deal with an important aspect when studying the complexity of network-based systems. In particular, we establish relations between information-theoretic complexity measures [3], [8]–[11]. Recall that such entropic measures have been used to quantify the information content of the underlying networks [8], [12]. Generally, this relates to exploring the complexity of a graph by taking its structural features into account. Note that numerous measures have been developed to study the structural complexity of graphs [5], [8], [13]–[22]. Further, the use and ability of the measures has been demonstrated by solving interdisciplinary problems. As a result, such studies have led to a vast number of contributions dealing with the analysis of complex systems by means of information-theoretic measures, see, e.g., [8], [13]–[22]. Figure 1 shows a classification scheme of quantitative network measures exemplarily.

The main contribution of this paper is to study relations between entropy measures. We will tackle this problem by means of inequalities involving network information measures. In particular, we study so-called *implicit information inequalities* which have been introduced by Dehmer et al. [23], [24] for studying graph entropies using information functionals. Generally, an implicit information inequality involves information measures which are present on either side of the inequality. It is important to emphasize that relatively little work has been done to investigate relations between network measures. A classical contribution in this area is due to Bonchev et al. [25]. Here, the relatedness between information-theoretic network measures has been investigated to detect branching in chemical networks. Further, implicit information inequalities have been studied for hierarchical graphs which turned out to be useful in network biology [26].

We first present closed form expressions of graph entropies using the graph classes, stars and path graphs. Further, we infer novel information inequalities for the measures based on the 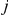-sphere functional. The section “Implicit Information Inequalities” presents our main results on novel implicit inequalities for networks. We conclude the paper with a summary and some open problems. Before discussing our results, we will first present the information-theoretic measures that we want to investigate in this paper.

## Methods

In this section, we briefly state the concrete definitions of the information-theoretic complexity measures that are used for characterizing complex network structures [3], [6], [9], [27]. Here we state measures based on two major classifications namely partition-based and partition-independent measures and deal mainly with the latter.

Given a simple, undirected graph 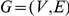, let 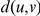 denote the distance between two vertices 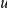 and 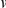, and let 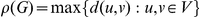. Let 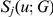 denote the 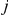-sphere of a vertex 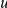 defined as 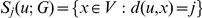. Throughout this article, a graph 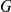 represents a simple undirected graph.

**Definition 1** *Let* 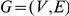 *be a graph on* 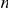 *vertices and let* 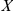 *be a graph invariant of* 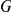. *Let* 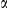 *be an equivalence relation that partitions* 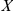 *into* 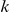 *subsets* 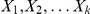, *with cardinality* 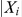 *for* 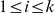. *The total structural information content of* 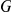 *is given by*

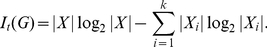
(1)


**Definition 2** *Let* 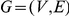 *be a graph on* 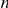 *vertices and let* 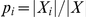, *for* 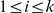 *be the probability value for each partition. The mean information content of* 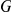 *is*

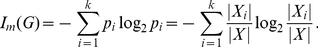
(2)


In the context of theory of communication, the above equation is called as Shannon equation of information [28].

**Definition 3** *Let* 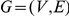 *be a graph on* 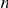 *vertices. The quantity*

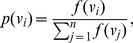
(3)
*is a probability value of* 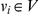. 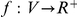 *is an arbitrary information functional that maps a set of vertices to the non-negative real numbers.*

**Remark 1** *Observe that,* 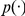 *defines a probability distribution over the set of vertices as it satisfies* 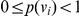, *for every vertex* 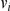, 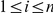 *and* 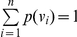.

Using the resulting probability distribution associated with 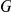 leads to families of network information measures [3], [9].

**Definition 4** *The graph entropy of* 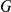 *given representing its structural information content:*

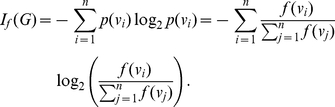
(4)


In order to define concrete graph entropies, we reproduce the definitions of some information functionals based on metrical properties of graphs [3], [9], [27].

**Definition 5** *Parameterized exponential information functional using* 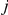 *-spheres:*

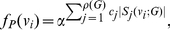
(5)where 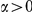 and 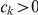 for 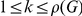.

**Definition 6** *Parameterized linear information functional using* 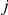 *-spheres:*

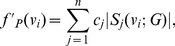
(6)where 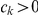 for 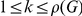.

**Remark 2** *Observe that, when either* 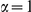 *or the* 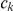 *are all equal, the functional* 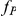 *and* 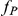 *becomes a constant function and, hence, the probability on all the vertices are equal. That is* 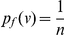, *for* 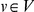. *Thus, the value of the entropy attains its maximum value,* 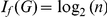. *Thus, in all our proofs, we only consider the non-trivial case, namely* 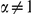 *and/or at least for two coefficients holds* 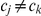.

Next, we will define the local information graph to use local centrality measures from [9]. Let 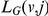 be the subgraph induced by the shortest path starting from the vertex 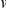 to all the vertices at distance 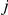 in 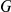. Then, 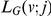 is called the *local information graph regarding* 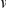 *with respect to* 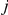, see [9]. A local centrality measure that can be applied to determine the structural information content of a network [9] is then defined as follows.

**Definition 7** *The closeness centrality of the local information graph is defined by*

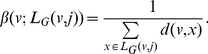
(7)


**Remark 3** *Note that centrality is an important concept that has been introduced for analyzing social networks* [29], [30] *. Many centrality measures have been contributed* [30] *, and in particular, various definitions for closeness centrality* [30]–[32] *. We remark that the above definition has been firstly defined by Sabidussi* [31] *for arbitrary graphs. However, we use the measure as a local invariant defined on the subgraphs induced by the local information graph* [9] *.*

Similar to the 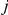-sphere functionals, we define further functionals based on the local centrality measure as follows.

**Definition 8** *Parameterized exponential information functional using local centrality measure:*

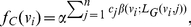
(8)where 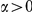, 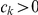 for 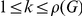.

**Definition 9** *Parameterized linear information functional using local centrality measure:*

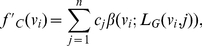
(9)where 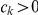, for 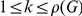.

Note that the coefficients 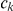 can be chosen arbitrarily. However, the functionals become more meaningful when we choose the coefficients to emphasize certain structural characteristics of the underlying graphs. Also, this remark implies that the notion of graph entropy is not unique because each measure takes different structural features into account. Further, this can be understood by the fact that a vast number of entropy measures have been developed so far. Importantly, we point out that the measures we explore in this paper are notably different to the notion of graph entropy introduced by Körner [21]. The graph entropy due to Körner [21] is rooted in information theory and based on the known stable set problem. To study more related work, survey papers on graph entropy measures have been authored by Dehmer et al. [3] and Simonyi [33].

## Results and Discussion

### Closed Form Expressions and Explicit Information Inequalities

When calculating the structural information content of graphs, it is evident that the determination of closed form expressions using arbitrary networks is critical. In this section, we consider simple graphs namely trees with smallest and largest diameter and compute the measures defined in the previous section. By using arbitrary connected graphs, we also derive explicit information inequalities using the measures based on information functionals (stated in the previous section).

#### Stars

Star graphs, 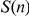, have been of considerable interest because they represent trees with smallest possible diameter (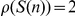) among all trees on 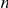 vertices.

Now, we present closed form expressions for the graph entropy by using star graphs. For this, we apply the information-theoretic measures based on information functionals defined in the preliminaries section.

**Theorem 4** *Let* 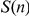 *be a star on* 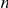 *vertices. Let* 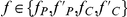 *be the information functionals as defined before. The information measure is given by*

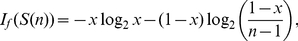
(10)
*where* 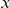 *is the probability of the central vertex of* 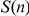 *:*

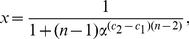
(11)if 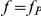.
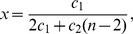
(12)if 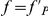.
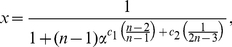
(13)if 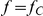.
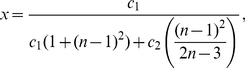
(14)if 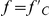.

**Proof:**

- Consider 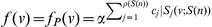, where 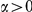 and 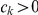 for 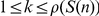.

We get,
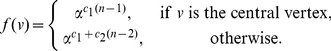
(15)Therefore,
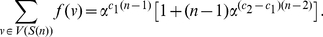
(16)Hence,
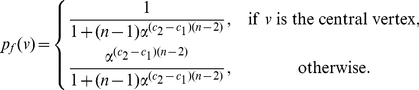
(17)By substituting the value of 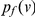 in 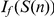 and simplifying, we get
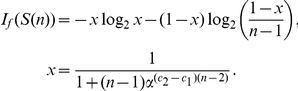

- Consider 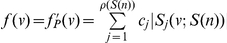, where 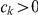 for 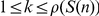.

We get,
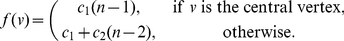
(18)Therefore,
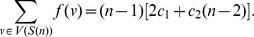
(19)Hence,
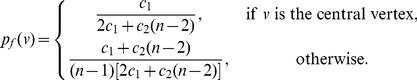
(20)By substituting the value of 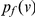 in 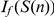 and simplifying, we get
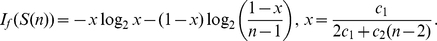

- Consider the case 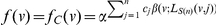, where 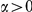, 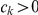 for 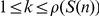.

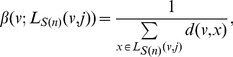
(21)denotes the closeness centrality measure.

Then, we yield
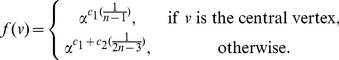
(22)Therefore,
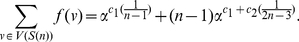
(23)Hence,
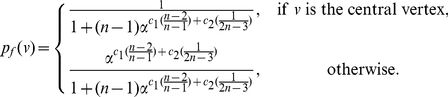
(24)By substituting the value of 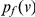 in 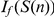 and simplifying, we obtain
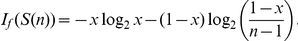
where 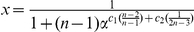.

- Consider 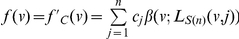, where 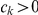 for 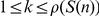. 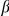 is defined via Equation (18). We get,
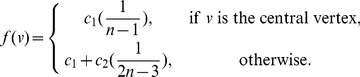
(25)Therefore,
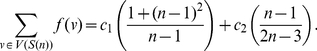
(26)Thus,
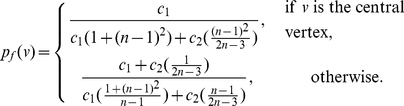
(27)By substituting the value of 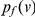 in 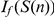 and simplifying, we get
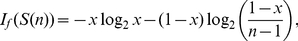
(28)where 
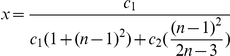
. 

By choosing particular values for the parameters involved, we get concrete measures using the above stated functionals. For example, consider the functional 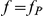 and set
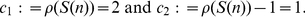
(29)If we plug in those values in Equations (10) and (11), we easily derive
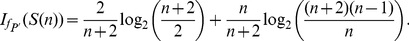
(30)


#### Paths

Let 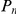 be the path graph on 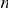 vertices. Path graphs are the only trees with maximum diameter among all the trees on 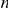 vertices, i.e., 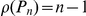. We remark that to compute a closed form expression even for path graphs, is not always simple. To illustrate this, we present the concrete information measure 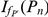 by choosing particular values for its coefficients.

**Lemma 5** *Let* 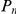 *be a path graph and consider the functional* 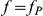 *defined by* *Equation (6)* *. We set* 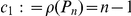, 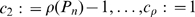. *We yield*

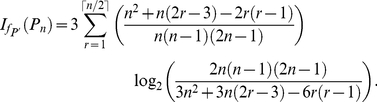
(31)


**Proof:** Let 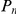 be a path graph trivially labeled by 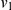, 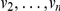 (from left to right).

Given 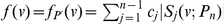 with 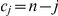 for 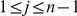.

By computing 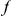, when 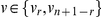, for 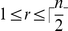, we infer
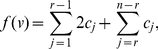
(32)

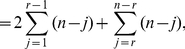
(33)

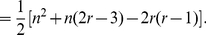
(34)Therefore,
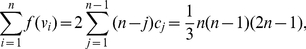
(35)and, hence,
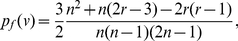
(36)where 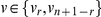, for 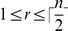. By substituting these quantities into 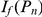 yields the desired result.

Note that when using the same measure with arbitrary coefficients, its computation is intricate. In this regard, we present explicit bounds or information inequalities for any connected graph if the measure is based on the information functional using 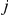-spheres. That is, either 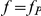 or 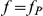.

#### General connected graphs

**Theorem 6** *Given any connected graph* 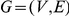 *on* 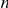 *vertices and let* 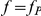 *given by* *Equation (5)* *. Then, we infer the following bounds:*

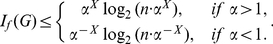
(37)

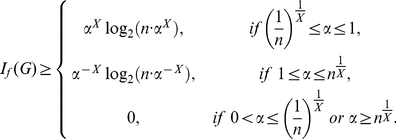
(38)

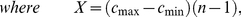
(38)

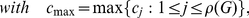
(40)

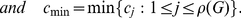
(41)


**Proof:** Consider 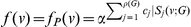, where 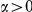 and 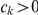 for 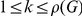. Let 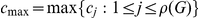 and 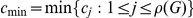. Recall (see Remark (2)) that, when either 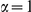 or when all the coefficients (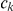) are equal, the information functional becomes constant and, hence, the value of 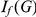 equals 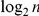. In the following, we will discuss the cases 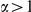 and 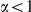, and we also assume that not all 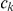 are equal.

Case 1: 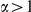: We first construct the bounds for 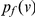 as shown below:
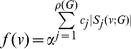
(42)

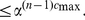
(43)Similarly,
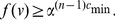
(44)Therefore, from the Equations (43) and (44), we get
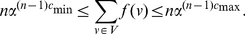
(45)Hence,
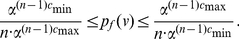
(46)Let 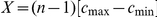. Then, the last inequality can be rewritten as,
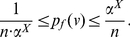
(47)


Upper bound for 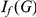:

Since 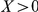 and 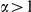, we have 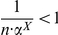. Hence, we have 
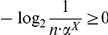
 and 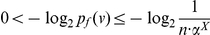. Thus we get,
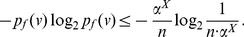
(48)By adding over all the vertices of 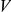, we obtain
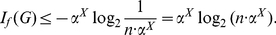
(49)


Lower bound for 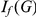:

We have to distinguish two cases, either 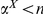 or 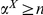.

Case 1.1: 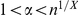. We yield 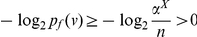. Therefore,
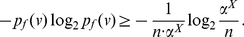
(50)By adding over all the vertices of 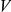, we get
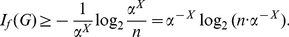
(51)


Case 1.2: 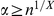.

In this case, we obtain 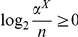 and 
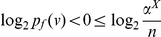
. Therefore, by using these bounds in Equation (4), we infer 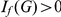.

Case 2: 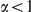:

Consider Equation (42). We get the following bounds for 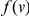:
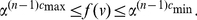
(52)Therefore,
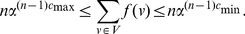
(53)Hence,
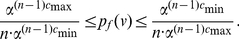
(54)Set 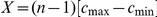. Then, the last inequality can be rewritten as,
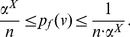
(55)


Upper bound for 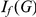:

Since 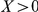 and 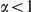, we have 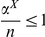. Hence, we have 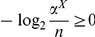 and 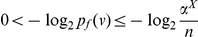. Thus, we obtain,
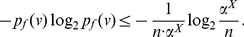
(56)By adding over all the vertices of 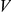, we get
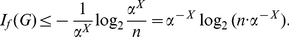
(57)


Lower bound for 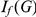:

Again, we consider two cases, either 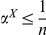 or 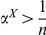.

Case 2.1: 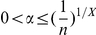.

In this case, we have 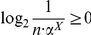 and 
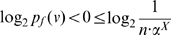
. Therefore, by substituting these bounds in the Equation (4), we obtain 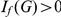.

Case 2.2: 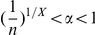.

We have 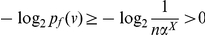. Therefore,
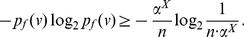
(58)By adding over all the vertices of 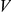, we get
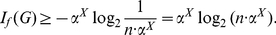
(59)Hence, the theorem follows.

In the next theorem, we obtain explicit bounds when using the information functional given by Equation (6).

**Theorem 7** *Given any connected graph* 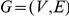 *on* 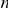 *vertices and let* 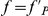 *be given as in* *Equation (6)* *. We yield*

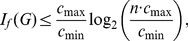
(60)

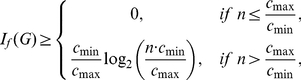
(61)

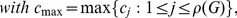
(62)

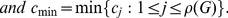
(63)


**Proof:** Consider 
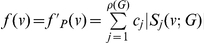
, where 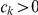 for 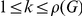. Let 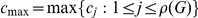 and 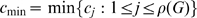. We have,
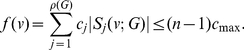
(64)Similarly,
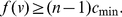
(65)Therefore, from the Equations (64) and (65), we get
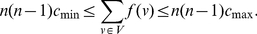
(66)Hence,
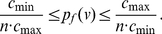
(67)


Upper bound for 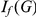:

Since 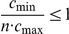, we have 
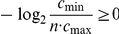
 and 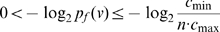. Hence,
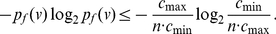
(68)By adding over all the vertices of 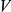, we obtain
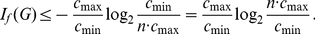
(69)


Lower bound for 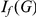:

Let us distinguish two cases:

Case 1: 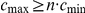.

We have 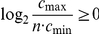 and 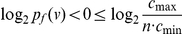. Therefore, by applying these bounds to Equation (4), we obtain 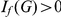.

Case 2: 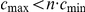.

In this case, we have 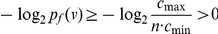. Therefore,
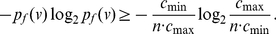
(70)By adding over all the vertices of 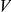, we obtain the lower bound for 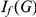 given by
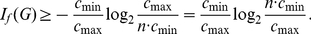
(71)Hence, the theorem follows.

### Implicit Information Inequalities

Information inequalities describe relations between information measures for graphs. An implicit information inequality is a special type of an information inequality where the entropy of the graph is estimated by a quantity that contains another graph entropy expression. In this section, we will present some implicit information inequalities for entropy measures based on information functionals. In this direction, a first attempt has been done by Dehmer et al. [23], [24], [26]. Note that Dehmer et al. [23], [26] started from certain conditions on the probabilities when two different information functionals 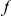 and 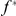 are given. In contrast, we start from certain assumptions which the functionals themselves should satisfy and, finally, derive novel implicit inequalities. Now, given any graph 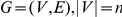. Let 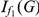 and 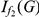 be two mean information measures of 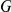 defined using the information functionals 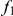 and 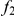 respectively. Let us further define another functional 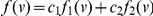, 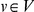. In the following, we will study the relation between the information measure 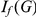 and the measures 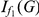 and 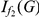.

**Theorem 8** *Suppose* 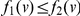, *for all* 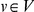, *then the information measure* 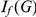 *can be bounded by* 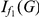 *and* 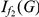 *as follows:*

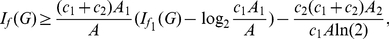
(72)

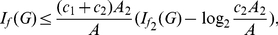
(73)where 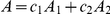, 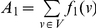, *and* 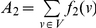.

**Proof:** Given 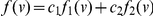. Let 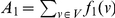 and 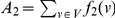. Therefore 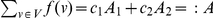. The information measures of 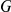 with respect to 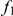 and 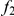 are given by
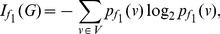
(74)where 
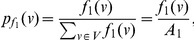


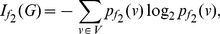
(75)where 
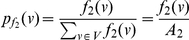
.

Now consider the probabilities,
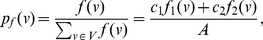
(76)

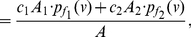
(77)

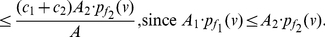
(78)Using Equation (77) and based on the fact that 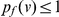, we get
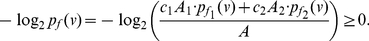
(79)Thus,
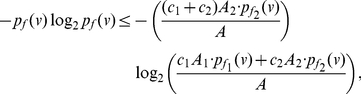
(80)and
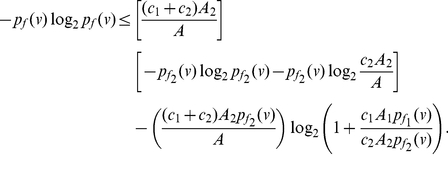
(81)Since the last term in the above inequality is positive, we get
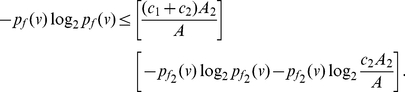
(82)By adding up the above inequalities over all the vertices of 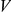, we get the desired upper bound. From Equation (77), we also get a lower bound for 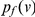, given by
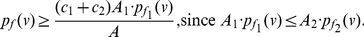
(83)Now proceeding as before with the above inequality for 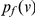, we obtain
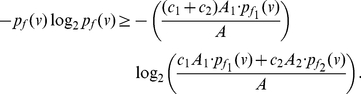
(84)

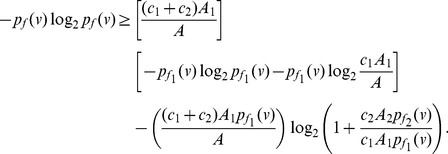
(85)By using the concavity property of the logarithm, that is, 
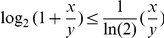
, we yield
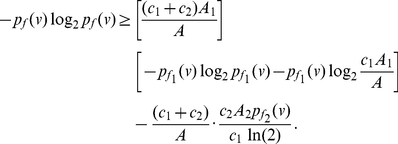
(86)By adding the above inequality over all the vertices of 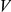, we get the desired lower bound. This proves the theorem.

**Corollary 9** *The information measure* 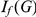, *for* 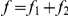, *is bounded by* 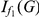 *and* 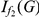 *as follows:*

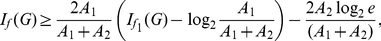
(87)

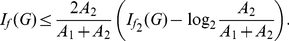
(88)


**Proof:** Set 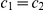 in Theorem (8), then the corollary follows.

**Corollary 10** *Given two information functionals,* 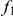, 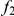 *such that* 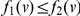, 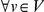. *Then*

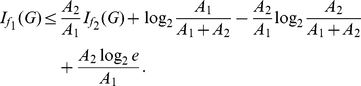
(89)


**Proof:** Follows from Corollary (9).

The next theorem gives another bound for 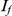 in terms of both 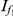 and 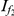 by using the concavity property of the logarithmic function.

**Theorem 11** *Let* 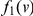 *and* 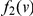 *be two arbitrary functionals defined on a graph* 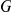. *If* 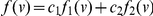 *for all* 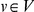, *we infer*

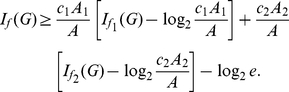
(90)
*and*

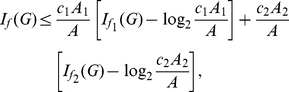
(91)
*where* 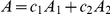, 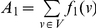 *and* 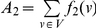.

**Proof:** Starting from the quantities for 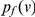 based on Equation (77), we obtain
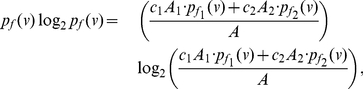
(92)

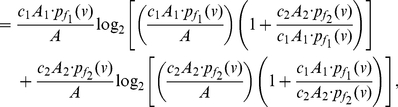
(93)

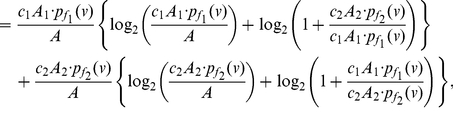
(94)

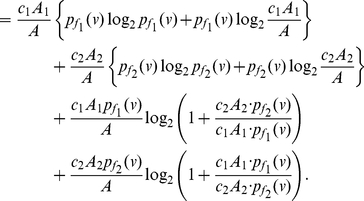
(95)Since each of the last two terms in Equation (95) is positive, we get a lower bound for 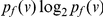, given by
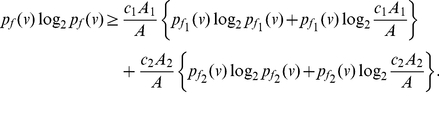
(96)Applying the last inequality to Equation (4), we get the upper bound as given in Equation (91). By further applying the inequality 
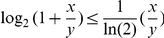
 to Equation (95), we get an upper bound for 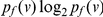, given by
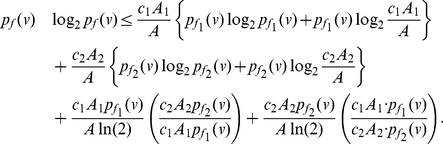
(97)Therefore,
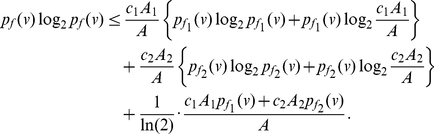
(98)Finally, we now apply this inequality to Equation (4) and get the lower bound as given in Equation (90).

The next theorem is a straightforward extension of the previous statement. Here, an information functional is expressed as a linear combination of 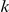 arbitrary information functionals.

**Theorem 12** *Let* 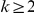 *and* 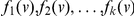 *be arbitrary functionals defined on a graph* 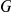. 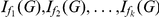 *are the corresponding information contents. If* 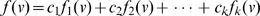 *for all* 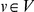, *we infer*

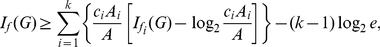
(99)
*and*

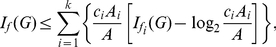
(100)
*where* 
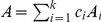
, 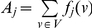 for 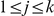. 


#### Union of Graphs

In this section, we determine the entropy of the union of two graphs. Let 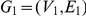 and 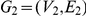 be two arbitrary connected graphs on 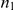 and 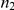 vertices, respectively. Let 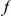 be an information functional defined on these graphs denoted by 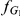, 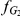 and let 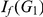 and 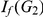 be the information measures on 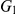 and 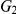 respectively.

**Theorem 13** *Let* 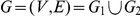 *be the disjoint union of the graphs* 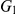 *and* 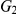. *Let* 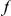 *be an arbitrary information functional. The information measure* 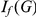 *can be expressed in terms of* 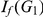 *and* 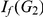 *as follows:*

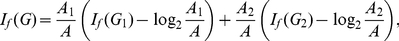
(101)
*where* 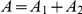 *with* 
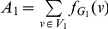
 *and* 
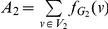
.

**Proof:** Let 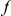 be the given information functional. Let 
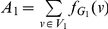
 and 
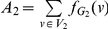
. The information measures of 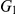 and 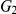 are given as follows:
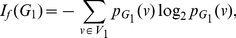
(102)where 
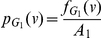
, and
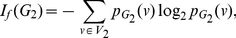
(103)

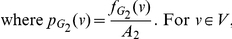


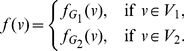
(104)Hence,
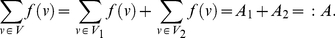
(105)

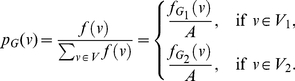
(106)

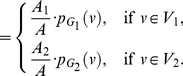
(107)Using these quantities to determine 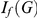, we obtain
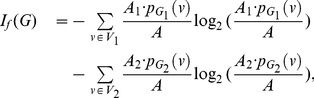
(108)and
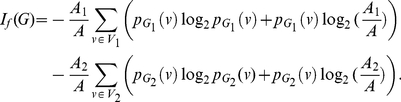
(109)Upon simplification, we get the desired result.

Also, we immediately obtain a generalization of the previous theorem by taking 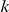-disjoint graphs into account.

**Theorem 14** *Let* 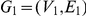, 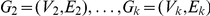 *be* 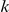 *arbitrary connected graphs on* 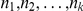 *vertices, respectively. Let* 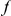 *be an information functional defined on these graphs denoted by* 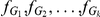. *Let* 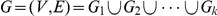 *be the disjoint union of the graphs* 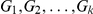 *for* 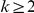. *The information measure* 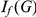 *can be expressed in terms of* 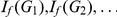, 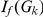 *as follows:*

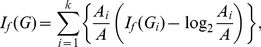
(110)
*where* 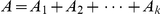 *with* 
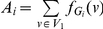
 *for* 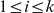.

#### Join of Graphs

Let 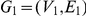 and 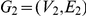 be two arbitrary connected graphs on 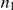 and 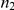 vertices, respectively. The join of the graphs 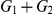 is defined as the graph 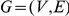 with vertex set 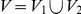 and the edge set 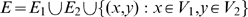. Let 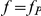 be the information functional (given by Equation (5)) based on the 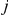-sphere functional (exponential) defined on these graphs and denoted by 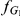, 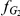. Let 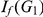 and 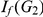 be the information measures on 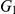 and 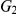 respectively.

**Theorem 15** *Let* 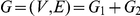 *be the join of the graphs* 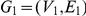 *and* 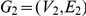 *with* 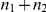 *vertices. The information measure* 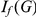 *can then be expressed in terms of* 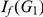 *and* 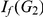 *as follows:*

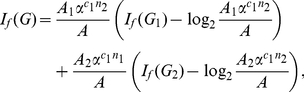
(111)
*where* 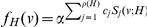 *for* 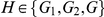, 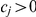 *and* 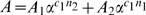 *with* 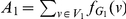 *and* 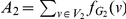.

**Proof:** Let 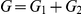 be the join of two connected graphs 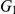 and 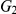. Here, 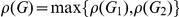. Let 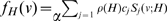 be the information functional defined by using the 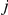-sphere functional on 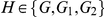. Let 
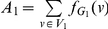
 and 
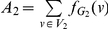
. The information measures of 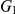 and 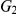 are given as follows:
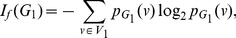
(112)

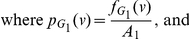


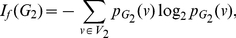
(113)

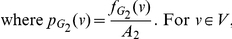


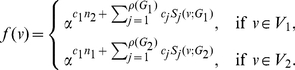
(114)

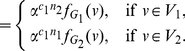
(115)Hence,
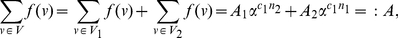
(116)

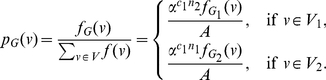
(117)

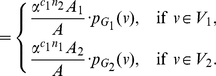
(118)Using those entities to determine 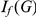, we infer
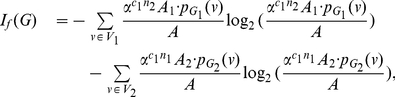
(119)and
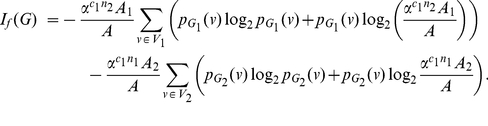
(120)Upon simplification, we get the desired result.

If we consider the linear 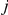-sphere functional 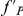 (see Equation (6)), to infer an exact expression for the join of two graphs as in Theorem (15) is an intricate problem. By Theorem (16) and Theorem (17), we will now present different bounds in terms of 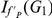 and 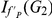.

**Theorem 16** *Let* 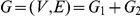 *be the join of the graphs* 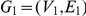 *and* 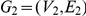 *on* 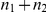 *vertices. Then, we yield*

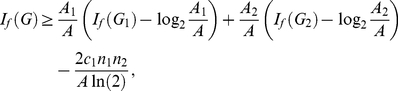
(121)
*where* 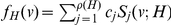 *for* 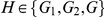, 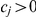 *and* 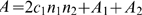 *with* 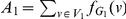 *and* 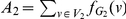.

**Proof:** Let 
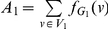
 and 
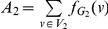
. The information measures of 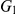 and 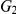 are given as follows:
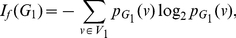
(122)

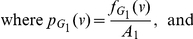


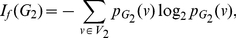
(123)

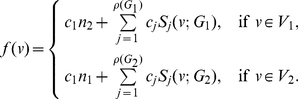
(124)

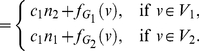
(125)Hence,
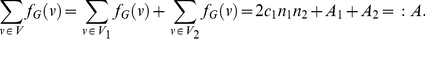
(126)

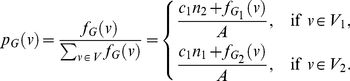
(127)

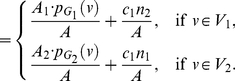
(128)Since 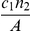 and 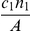 are positive, we get a lower bound for 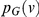 given as
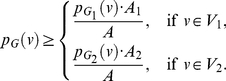
(129)To infer a lower bound for the information measure 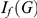, we start from the Equations (128), (129) and obtain
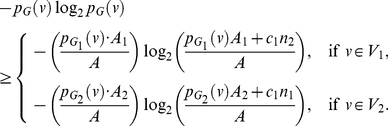
(130)

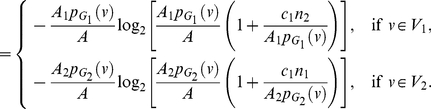
(131)

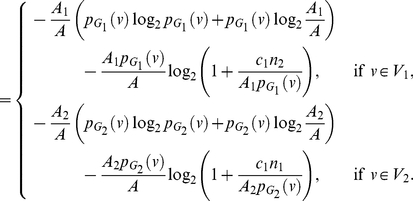
(132)By using the inequality 
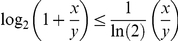
 and performing simplification steps, we get,
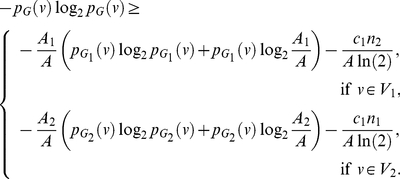
(133)By adding up the above inequality system (across all the vertices of 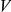) and by simplifying, we get the desired lower bound.

Further, an alternate set of bounds can be achieved as follows.

**Theorem 17** *Let* 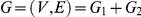 *be the join of the graphs* 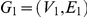 *and* 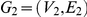 *on* 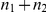 *vertices. Then, we infer*

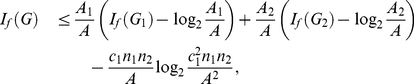
(134)
*and*

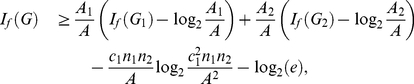
(135)
*where* 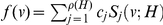 *for* 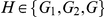, 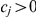 *and* 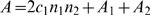 *with* 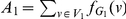 *and* 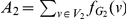.

**Proof:** Starting from Theorem (16), consider the value of 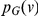 given by Equation (128). By using the quantities for 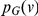 to calculate 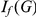, we get
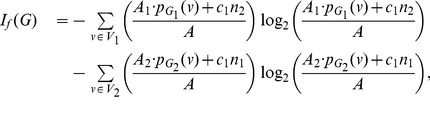
(136)and
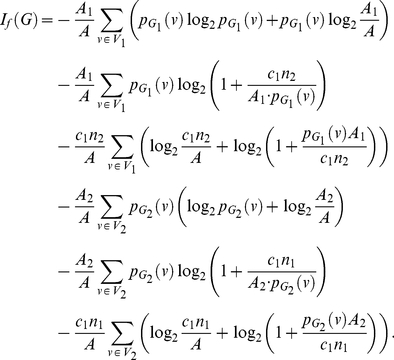
(137)By simplifying and performing summation, we get
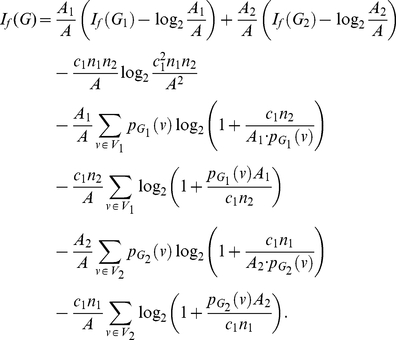
(138)An upper bound for the measure 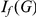 can be derived as follows:
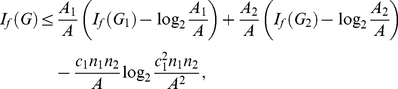
(139)since each of the remaining terms in Equation (138) is positive. Finally, we infer the lower bound for 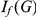 as follows. By applying inequality 
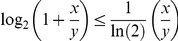
 to Equation (138), we get
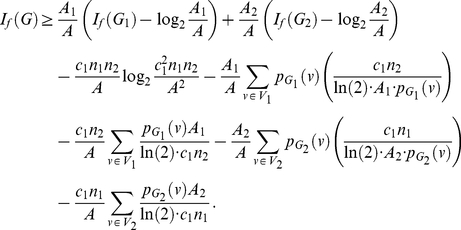
(140)Upon simplification, we get
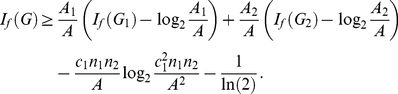
(141)Putting Inequality (139) and Inequality (141) together finishes the proof of the theorem.

### Summary and Conclusion

In this article, we have investigated a challenging problem in quantitative graph theory namely to establish relations between graph entropy measures. Among the existing graph entropy measures, we have considered those entropies which are based on information functionals. It turned out that these measures have widely been applicable and useful when measuring the complexity of networks [3].

In general, to find relations between quantitative network measures is a daunting problem. The results could be used in various branches of science including mathematics, statistics, information theory, biology, chemistry and social sciences. Further, the determination of analytical relations between measures is of great practical importance when dealing with large scale networks. Also, relations involving quantitative network measures could be fruitful when determining the information content of large complex networks.

Note that our proof technique follows the one proposed in [23]. It is based on three main steps: Firstly, we compute the information functionals and in turn, we calculate the probability values for every vertex of the graph in question. Secondly, we start with certain conditions for the computed functionals and arrive at a system of inequalities. Thirdly, by adding up the corresponding inequality system, we obtain the desired implicit information inequality. Using this approach, we have inferred novel bounds by assuming certain information functionals. It is evident that further bounds could be inferred by taking novel information functionals into account. Further, we explored relations between the involved information measures for general connected graphs and for special classes of graphs such as stars, path graphs, union and join of graphs.

At this juncture, it is also relevant to compare the results proved in this paper with those proved in [23]. While we derived the implicit information inequalities by assuming certain properties for the functionals, the implicit information inequalities derived in [23] are based on certain conditions for the calculated vertex probabilities. Interestingly, note that by using Theorem (11) and Theorem (17), the range of the corresponding bounds is very small. We inferred that the difference between the upper and lower bound equals 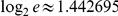.

As noted earlier, relations between entropy-based measures for graphs have not been extensively explored so far. Apart from the results we have gained in this paper, we therefore state a few open problems as future work:

- To find relations between 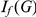 and 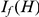, when 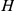 is an induced subgraph of 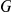 and 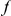 is an arbitrary information functional.
- To find relations between 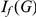 and 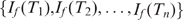, where 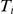, 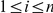 are so-called generalized trees, see [34]. Note that it is always possible to decompose an arbitrary, undirected graph into a set of generalized trees [34].
- To find relations between measures based on information functionals and the other classical graph measures.
- To derive information inequalities for graph entropy measures using random graphs.
- To derive statements to judge the quality of information inequalities.

## References

[1] Basak SC, Devillers J, Balaban AT (1999). Information-theoretic indices of neighborhood complexity and their applications.. Topological Indices and Related Descriptors in QSAR and QSPAR, Gordon and Breach Science Publishers.

[2] Wang J, Provan G, Zhou J (2009). Characterizing the structural complexity of real-world complex networks..

[3] Dehmer M, Mowshowitz A (2011). A history of graph entropy measures.. Information Sciences.

[4] Li M, Vitányi P (1997). An Introduction to Kolmogorov Complexity and Its Applications.

[5] Mowshowitz A (1968). Entropy and the complexity of graphs: I. an index of the relative complexity of a graph.. Bulletin of Mathematical Biophysics.

[6] Shannon CE (1948). A mathematical theory of communication.. Bell System Technical Journal.

[7] Bonchev D, Rouvray DH (2003). Complexity in chemistry: Introduction and Fundamentals. Mathematical and Computational Chemistry 7.

[8] Bonchev D (1983). Information Theoretic Indices for Characterization of Chemical Structures..

[9] Dehmer M (2008). Information processing in complex networks: graph entropy and information functionals.. Appl Math Comput.

[10] Emmert-Streib F, Dehmer M (2007). Information theoretic measures of UHG graphs with low computational complexity.. Appl Math Comput.

[11] Mehler A, Weiß P, Lücking A (2010). A network model of interpersonal alignment.. Entropy.

[12] Bonchev D (2003). Complexity in Chemistry..

[13] Anand K, Bianconi G (2009). Entropy measures for networks: Toward an information theory of complex topologies.. Phys Rev E.

[14] Costa LdF, Rodrigues FA, Travieso G, Boas PRV (2007). Characterization of complex networks: A survey of measurements.. Advances in Physics.

[15] Kim J, Wilhelm T (2008). What is a complex graph?. Physica A: Statistical Mechanics and its Applications.

[16] Balaban AT, Balaban TS (1991). New vertex invariants and topological indices of chemical graphs based on information on distances.. Journal of Mathematical Chemistry.

[17] Bertz SH, King R (1983). A mathematical model of complexity..

[18] Basak SC, Magnuson VR, Niemi GJ, Regal RR (1988). Determining structural similarity of chemicals using graph-theoretic indices.. Discrete Applied Mathematics.

[19] Bonchev D, Rouvray DH (2005). Complexity in chemistry, biology, and ecology. Mathematical and Computational Chemistry.

[20] Claussen JC (2007). Offdiagonal complexity: A computationally quick complexity measure for graphs and networks.. Physica A: Statistical Mechanics and its Applications.

[21] Körner J (1973). Coding of an information source having ambiguous alphabet and the entropy of graphs..

[22] Butts C (2001). The complexity of social networks: Theoretical and empirical findings.. Social Networks.

[23] Dehmer M, Mowshowitz A (2010). Inequalities for entropy-based measures of network information content.. Applied Mathematics and Computation.

[24] Dehmer M, Mowshowitz A, Emmert-Streib F (2011). Connections between classical and parametric network entropies.. PLoS ONE.

[25] Bonchev D, Trinajstic N (1977). Information theory, distance matrix, and molecular branching.. The Journal of Chemical Physics.

[26] Dehmer M, Borgert S, Emmert-Streib F (2008). Entropy bounds for hierarchical molecular networks.. PLoS ONE.

[27] Skorobogatov VA, Dobrynin AA (1988). Metrical analysis of graphs.. MATCH Commun Math Comp Chem.

[28] Shannon C, Weaver W (1997). The Mathematical Theory of Communication..

[29] Freeman LC (1977). A set of measures of centrality based on betweenness.. Sociometry.

[30] Freeman LC (1978). Centrality in social networks conceptual clarification.. Social Networks.

[31] Sabidussi G (1966). The centrality index of a graph.. Psychometrika.

[32] Emmert-Streib F, Dehmer M (2011). Networks for systems biology: Conceptual connection of data and function.. IET Systems Biology.

[33] Simonyi G, Cook W, Lovász L, Seymour P (1995). Graph entropy: A survey..

[34] Emmert-Streib F, Dehmer M, Kilian J, et al HRA, editor (2006). Classification of large graphs by a local tree decomposition..

